# Association Between Cytomegalovirus and Epstein-Barr Virus Co-Reactivation and Hematopoietic Stem Cell Transplantation

**DOI:** 10.3389/fcimb.2022.818167

**Published:** 2022-03-25

**Authors:** Shan-shan Li, Na Zhang, Mei Jia, Ming Su

**Affiliations:** Department of Clinical Laboratory, Peking University People’s Hospital, Beijing, China

**Keywords:** co-reactivation, CMV, EBV, HSCT, outcomes

## Abstract

The co-reactivation of cytomegalovirus (CMV) and Epstein-Barr virus (EBV) in patients undergoing hematopoietic stem cell transplantation (HSCT) has been found. Research has shown that the reactivation of CMV or EBV is closely related to poor HSCT outcomes. In this study, we describe the clinical characteristics of HSCT patients with co-reactivation of CMV and EBV. We retrospectively reviewed the medical records of 327 patients who underwent HSCT at the Peking University People’s Hospital Institute of Hematology. Co-reactivation of CMV and EBV was observed in a total of 75 patients (22.9%) who also had a higher incidence of hemorrhagic cystitis (P=0.000). HSCT patients with CMV and co-reactivation of CMV and EBV had a significantly lower 1-year overall survival (OS; P=0.050). Further, COX regression analysis showed that viral infection was a risk factor for 1-year OS (HR, 12.625 for co-reactivation *vs*. no reactivation, p=0.021, and HR 13.580 for CMV reactivation *vs*. no reactivation, P=0.013). In conclusion, the patients with CMV reactivation had poorer outcome after HSCT regardless of EBV reactivation.

## Introduction

Despite advanced treatment and prevention measures, double-stranded DNA (dsDNA) viruses can still increase the mortality of patients treated with allogeneic hematopoietic stem cell transplantation (HSCT). The reactivation of multiple herpes viruses, especially cytomegalovirus (CMV) reactivation, commonly occurs following HSCT (Hill et al., 2017; Stern et al., 2021). Epstein-Barr virus (EBV) reactivation is also prevalent and can cause serious complications, such as post-transplant lymphoproliferative disorder (PTLD) (Diop et al., 2021; Enok Bonong et al., 2021). The reported incidences of virus reactivation after HSCT fluctuate widely from 0.1% to 63% for EBV (Styczynski et al., 2016b) and from 30% to 70% for CMV (Styczynski et al., 2016a; Ljungman et al., 2019) with ambiguous effects on transplant outcomes (Auger et al., 2014; Teira et al., 2016).

CMV and EBV are the most closely related clinical viruses with a clear therapeutic association (Einsele et al., 2020; Yoshimori et al., 2021). There seems to be a bidirectional relationship between the two viruses, and a high incidence/low clearance rate of CMV infection and a high incidence and delayed immune reconstitution of EBV-PTLD are key to all of these findings (Green et al., 2013; Chiereghin et al., 2019). Therefore, we speculate that the co-activation of CMV and EBV after HSCT might lead to worse clinical outcomes in transplant patients compared to those with single virus reactivation. However, few studies have investigated the effects of CMV and EBV co-reactivation following HSCT. Our study analyzed the clinical characteristics of HSCT patients who developed co-reactivation of CMV and EBV, investigated the influence of CMV and EBV co-reactivation on HSCT outcomes, and analyzed the underlying risk factors.

## Materials and Methods

### Study Population

In total, 327 patients who underwent allo-HSCT for the first time between January 2018 and January 2019 at the Peking University People’s Hospital Institute of Hematology were included in our study. We performed a retrospective review of their medical records. The Ethics Committee of the Peking University People’s Hospital approved our study. Moreover, informed consent was waived owing to the retrospective nature of this study.

### Transplantation Procedure

The transplantation process is described as follows. The adjustment therapy was modified rabbit anti-thymocyte globulin (2.5 mg/kg/day; Sang Stat, Lyon, France) plus busulfan/cyclophosphamide (BU/CY; busulfan, 9.6 mg/kg i.v., given as 12 doses on days −8 to−-6) and cyclophosphamide, 1.8 g/m^2^/day i.v. on days −5 to −4) for the unrelated donor (URD) HSCT and haplo-HSCT groups (Wang et al., 2011) and modified BU/CY for the identical sibling donor (ISD) patients. The ISD and haplo-HSCT patients were given mobilized granulocyte colony-stimulating factor (G-CSF), which primed peripheral blood stem cells (PBSCs). The URD HSCT patients were given unmanipulated PBSCs (3.0 × 10^8^ cells per kg) and fresh G-CSF-mobilized (5 μg kg^−1^ daily for 5–6 days). To prevent graft-versus-host disease (GVHD), all patients were given mycophenolate mofetil, cyclosporine, and short-term methotrexate (Wang, Liu, Xu, Liu, Chen, Chen, Han, Shi and Huang 2011).

### Virus Therapy and Monitoring

CMV and EBV reactivation was tested twice per week using plasma samples with real-time quantitative polymerase chain reaction (PCR). All patients were given ganciclovir between days −9 and −2 (Huang et al., 2009). Pre-emptive therapy with either intravenous foscarnet (90 mg/kg/day) or intravenous ganciclovir (5 mg/kg, twice daily) was started when the patients had confirmed CMV viremia reactivation, and therapy was continued until the CMV DNA was not detected on two consecutive tests. Antiviral drugs, such as foscarnet, were given to patients with EBV reactivation. Moreover, rituximab was administered when EBV viremia persisted or deteriorated to EBV disease. The salvage therapy was EBV-specific CTL.

### Definitions

Our study defined myeloid engraftment as the first day of three continuous days with an absolute neutrophil count ≥0.5×10^9^/L, and platelet engraftment was recognized as the first day of seven consecutive days with a platelet count ≥20×10^9^/L without blood transfusion. Viral pneumonia mainly includes lung infections caused by influenza A, influenza B, paramyxoviruses, CMV, EBV, respiratory syncytial virus, adenovirus, and rhinoviruses. CMV and EBV reactivation were defined as the first of two continuous viral DNA tests in which the viral DNA reached or surpassed 1,000 copies/mL and 500 copies/mL, respectively. Co-reactivation of CMV and EBV was defined as a test of the EBV load and CMV load over 1 year after HSCT. The duration of viremia was defined as the number of days between the first day of viremia and the first day when the virus load was no longer detected Time to relapse was defined as the days between the date of HSCT and the date of disease relapse. Non-relapse mortality (NRM) was defined death as from all causes other than those directly related to the blood disease itself, occurring at any time after transplantation. Overall survival (OS) was defined as the days from HSCT to death from any cause. Leukemia-free survival (LFS) was defined as the days from HSCT to disease progression after HSCT.

### Statistical Analyses

Categorical variables between the groups were compared using the χ^2^ test or Fisher’s exact test. Continuous variables were compared using the Mann-Whitney U test. Multivariate Cox congress models were applied to test the hazards assumption and nonlinearity of the time-by-covariate interaction. The competing risk model was suggested to calculate the cumulative incidence of virus reactivation. IBM SPSS 25.0 statistical software (IBM SPSS Statistics, USA) and SAS (version 9.4; SAS institute Inc) were used in this research.

## Results

### Patient Characteristics

In total, 327 patients participated in this study. Patient characteristics are shown in **Tables 1** and **2**. Of these patients, 193 (59.0%) were men. The median age was 33 (25, 44) years. The majority of patients had acute myeloid leukemia (n=154, 47.1%) and acute lymphoblastic leukemia (n=102, 31.2%). Two hundred and forty-one (73.7%) patients received HSCT from haploidentical donors and 12 (3.7%) underwent HSCT originating from unrelated donors. Myeloid and platelet engraftments were found in 324 (99.1%) patients at a median of 13 (12–16) days and in 212 (64.8%) patients at a median of 15 (12– 20) days after HSCT, respectively. The morbidity of grade 1–2 acute GVHD and total acute GVHD was 32.4% (n=106) and 50.6% (n=119), respectively. The 1-year OS and LFS rates were 88.3% and 75.7%, respectively. The NRM and relapse rates causing mortality were 7.0% and 3.0%, respectively.

**Table 1:** Characteristics of patients.

Characteristics	Total	Co-reactivation group	CMV reactivation group	EBV reactivation group	No reactivation group	P value
**No. of patients (%)**	327	75 (22.9)	154 (47.1)	11 (3.3)	87 (26.6)	
**Gender no. (%)**						0.043
Male,	193 (59.0)	41 (54.7)	97 (63.0)	10 (90.9)	45 (51.7)	
Female	134 (41.0)	34 (45.3)	57 (37.0)	1 (9.1)	42 (48.3)	
**Age, median (rang)**	33 (25,44)	30 (24,37)	32 (25,43)	30 (24,44)	37 (29,48)	0.016
**Underlying disease, no. (%)**						0.563
AML	154 (47.1)	31 (41.3)	70 (45.4)	7 (63.6)	46 (52.9)	
ALL	102 (31.2)	27 (36.0)	50 (32.6)	4 (36.4)	21 (24.1)	
MDS	39 (11.9)	9 (12.0)	17 (11.0)	0	13 (14.9)	
Others*	32 (9.8)	8 (10.7)	17 (11.0)	0	7 (8.0)	
**Donor-recipient relationship, no. (%)**						<0.001
Mother/Father	123 (37.6)	47 (62.7)	55 (35.7)	7 (63.6)	14 (16.1)	
Son/Daughter	58 (17.8)	10 (13.3)	33 (21.4)	2 (18.2)	13 (14.9)	
Sibling	129 (39.4)	15 (20.0)	56 (36.4)	2 (18.2)	56 (64.4)	
Cousin	5 (1.5)	1 (1.3)	3 (1.9)	0	1 (1.2)	
Unrelated donor	12 (3.7)	2 (2.7)	7 (4.6)	0	3 (3.4)	
**HLA match, no. (%)**						<0.001
Haploidentical	241 (73.7)	69 (92.0)	126 (81.8)	10 (90.9)	36 (41.4)	
Identical	74 (22.6)	4 (5.3)	21 (13.6)	1 (9.1)	48 (55.2)	
Unrelated donor	12 (3.7)	2 (2.7)	7 (4.6)	0	3 (3.4)	
**Donor-Recipient gender**						0.376
Identical	159 (48.6)	42 (56.0)	74 (48.1)	6 (54.5)	37 (42.5)	
Different	168 (51.4)	33 (44.0)	80 (51.9)	5 (45.5)	50 (57.5)	
**ABO match**						0.715
matched	200 (61.2)	50 (66.7)	92 (59.7)	6 (54.5)	52 (59.8)	
mismatched	127 (38.8)	25 (33.4)	62 (40.3)	5 (45.5)	35 (40.2)	
**Time from diagnosis to transplantation**						0.408
Less than 1 year	281 (85.9)	64 (85.3)	129 (83.8)	11 (100.0)	77 (88.5)	
More than 1 year	46 (14.1)	11 (14.7)	25 (16.2)	0	10 (11.5)	
**Disease status**						0.621
CR1	288 (88.1)	64 (85.3)	134 (87.0)	11 (100.0)	79 (90.8)	
CR2 or NR	8 (2.4)	3 (4.0)	4 (2.6)	0	1 (1.2)	
others	31 (9.5)	8 (10.7)	16 (10.4)	0	7 (8.0)	
**MNCs in transplant (×10^8^/kg)**	8.65 (7.60,10.1)	8.88 (7.89,10.38)	8.85 (7.60,10.32)	8.42 (7.29,10.35)	8.19 (7.20,9.40)	0.022
**CD34+ cells in transplant (×10^6^/kg)**	2.27 (1.55,3.27)	1.87 (1.30,2.95)	2.46 (1.61,3.47)	2.39 (1.91,3.01)	2.28 (1.66,3.27)	0.059

Others* underlying diseases include aplastic anemia (18 patients), chronic myeloid leukemia (13 patients), and acute undifferentiated cell leukemia (one patient).

**Table 2:** Effect of CMV and EBV reactivation on clinical outcomes.

Clinical outcomes	Co-reactivation group	CMV reactivation group	EBV reactivation group	No reactivation group	P value
**Neutrophil engraftment**					
Planted (days)	13 (11,14)	13 (12,16)	13 (12,19)	14 (12,17)	0.010
Planted (%)	75 (100)	154 (100)	11 (100)	84 (97)	0.375
**Platelet engraftment**					
Planted (days)	15 (12,22)	15 (12,20)	20 (15,33)	13 (12,17)	0.080
planted (%)	46 (61.3)	98 (63.6)	6 (54.5)	62 (71.3)	0.460
**GVHD no. (%)**	48 (64.0)	109 (70.1)	9 (81.8)	54 (62.1)	0.340
a GVHD **no. (%)**	27 (56.3)	67 (61.5)	5 (55.6)	20 (37.0)	0.005
Grade I–II	24 (88.9)	58 (53.2)	5 (100.0)	19 (95.0)	0.544
Grade III–IV	3 (11.1)	9 (46.8)	0	1 (5.0)	
c GVHD **no. (%)**	17 (35.4)	31 (28.4)	3 (33.3)	32 (59.3)	
**CMV viremia**					
Time of first CMV viremia (days)	40 (31,47)	40 (33,49)	—	—	0.370
Duration of CMV viremia (days)	21 (15,26)	18 (11,24)	—	—	0.007
Highest CMV viral load, ×10^3^copies/m	1.71 (1.14,3.68)	1.69 (0.77,3.50)	—	—	0.315
**EBV viremia**					
Time of first EBV viremia (days)	50 (41, 66)	—	45 (41, 51)	—	0.280
Duration of EBV viremia (days)	13 (4, 20)	—	11 (4, 25)	—	0.916
Highest EBV viral load, ×10^3^copies/m	5.08 (1.43,6.60)	—	2.51 (1.38,3.16)	—	0.328
**Hemorrhagic cystitis, no. (%)**	28 (37.3)	54 (35.1)	3 (27.3)	10 (11.5)	<0.001
**Viral pneumonitis, no. (%)**	3 (4.0)	7 (4.5)	0	1 (1.1)	0.484
**Viral enteritis, no. (%)**	2 (2.7)	2 (1.3)	0	0	0.473
**Overall survival in 1 year after HSCT no. (%)**	65 (86.7)	131 (85.1)	10 (90.0)	83 (95.4)	0.050
**Leukemia free survival in 1 year after HSCT no. (%)**	53 (79.1)	101 (70.6)	10 (90.0)	66 (78.5)	0.112
**Mortality cause, no. (%)**					0.960
NRM	6 (8.0)	15 (9.7)	0	2 (2.3)	
Relapse	3 (4.0)	6 (4.0)	0	1 (1.1)	

### CMV and EBV Virus Reactivation

CMV viremia was found in 70.0% of the patients (n=229), among which, 47 patients were infected by CMV twice or more during 1 year after HSCT. The median time of the first CMV viremia reactivation after HSCT was 40 (33, 47) days, and the median duration was 18 (13, 25) days. The median CMV DNA copy number in patients was 2.27×10^3^ (1.42, 4.30×10^3^). Eighty-six patients (26.3%) had EBV reactivation. EBV viremia was reactivated at a median of 49 (41, 63) days after HSCT and lasted for a median of 13 (5, 20) days. EBV DNA copies reached 5.10×10^3^ (1.66, 2.62×10^3^). According to the aforementioned definition, 75 (22.9%) patients were classified as having co-reactivation of CMV and EBV. In total, 154 (47.1%) patients had CMV reactivation only and 87 (26.6%) patients had no reactivation of either virus (**Tables 1**, **2**).

### Co-Reactivation of CMV and EBV Affects Clinical Outcomes

Patients were divided into four groups according to CMV and EBV reactivation based on the following definitions: (1) co-reactivation group, (2) CMV reactivation group, (3) EBV reactivation group, and (4) no reactivation group. The characteristics of the four groups are presented in **Table 1**. Neutrophil engraftment was comparable among the four groups (100% *vs*. 100% *vs*. 100% *vs*. 97.0% for the co-reactivation, CMV reactivation, EBV reactivation, and no reactivation groups, respectively, P=0.375). However, neutrophil engraftment seemed to be delayed in patients with no virus reactivation, in contrast to that in the other groups (13 *vs*. 13 *vs*. 13 *vs*.14 days for co-reactivation, CMV reactivation, EBV reactivation, and no reactivation, respectively, P=0.010). According to platelet engraftment, the ratio of patients (61.3% *vs*. 63.6% *vs*. 54.5% *vs*. 71.3% for co-reactivation, CMV reactivation, EBV reactivation, and no reactivation, respectively, P=0.460) and days of engraftment (15 *vs*. 15 *vs*. 20 *vs*.13 days for co-reactivation, CMV reactivation, EBV reactivation, and no reactivation, respectively, P=0.080) were comparable among the four groups. The morbidity of acute GVHD was very higher in the reactivation group compared to that in the no reactivation group (56.3% *vs*. 61.5% *vs*. 55.6% *vs*. 37.0% for co-reactivation, CMV reactivation, EBV reactivation, and no reactivation, respectively, P=0.005). Patients in the reactivation group were more likely than those in the other groups to progress to viral pneumonia (4.0% *vs*. 4.5% *vs*. 0% *vs*. 1.1% for co-reactivation, CMV reactivation, EBV reactivation, and no reactivation, respectively, P=0.484), and a similar trend was observed for viral enteritis (2.7% *vs*. 1.3% *vs*. 0% *vs*. 0% for co-reactivation, CMV reactivation, EBV reactivation, and no reactivation, respectively, P=0.473). Hemorrhagic cystitis was more common in the reactivation group than in the other groups (37.5% *vs*. 35.2% *vs*. 14.1% *vs*. 14.1% for co-reactivation, CMV reactivation, EBV reactivation, and no reactivation, respectively, P=0.000, **Table 2**).

Co-reactivation and CMV reactivation were found in 22.9% (n=75) and 47.1% (n=154) of patients, respectively. In the co-reactivation group, the time of first CMV viremia reactivation after HSCT preceded that in the CMV reactivation group (40 [31, 47] *vs*. 40 [33, 49] days, respectively, P=0.370). The duration of CMV viremia in the co-reactivation group was longer than that in the CMV reactivation group (21 [15,26] *vs*.18 [11,24], days, respectively, P=0.007). The highest CMV viral load in the CMV reactivation group was higher than that in the CMV reactivation group (1.71 [1.14,3.68] *vs*.1.69 [0.77,3.50] ×10^3^ copies/mL, respectively, P=0.315). EBV reactivation was observed in 3.3% of the patients (n=11). The differences in the time of first EBV viremia reactivation after HSCT and the duration of EBV viremia were not statistically different between the two groups. Moreover, the highest EBV viral load was also not statistically different between the two groups.

**Figure 1:**
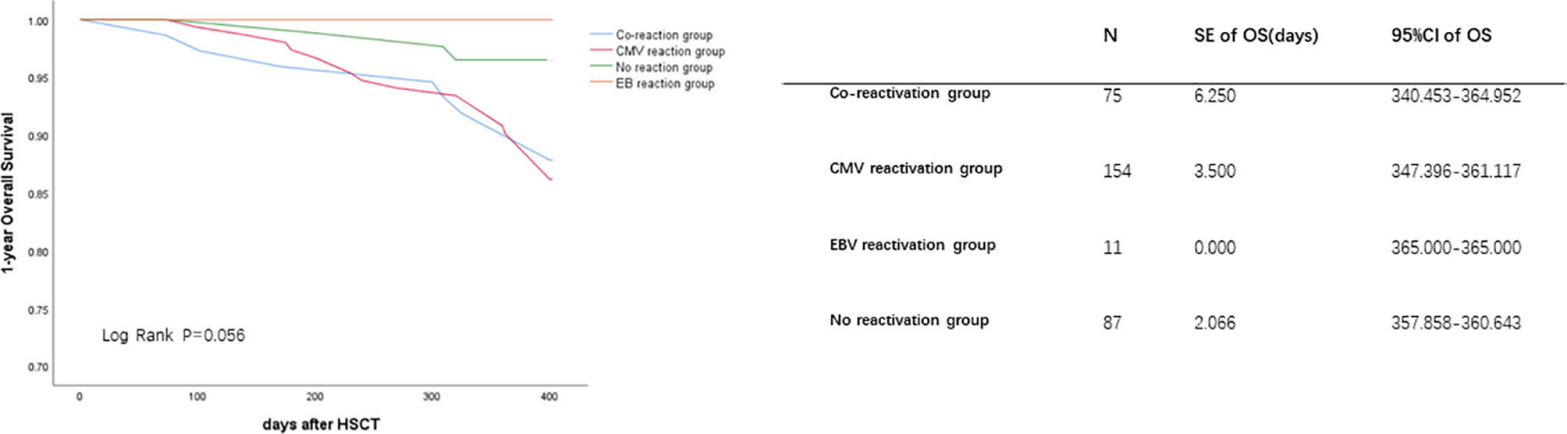
Cytomegalovirus (CMV) and Epstein-Barr virus (EBV) co-reactivation was identified as one of the risk factors for 1-year OS. The OS were performed by K-M method. *P* value was determined by Log-rank test. OS, overall survival; SE, standard error; CI, confidence interval.

**Figure 2:**
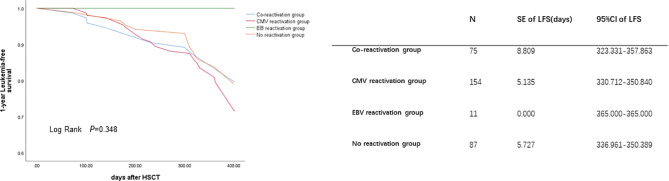
Cytomegalovirus (CMV) and Epstein-Barr virus (EBV) co-reactivation was identified as one of the risk factors for 1-year LFS. The LFS were performed by K-M method. *P* value was determined by Log-rank test. LFS, leukemia free survival; SE, standard error; CI, confidence interval.

The 1-year OS was lower in the reactivation groups than in the no reactivation group (86.7% *vs*. 85.1% *vs*. 90.0% *vs*. 95.4% for co-reactivation, CMV reactivation, EBV reactivation, and no reactivation, respectively, P=0.050). There were no significant differences in 1-year LFS among these groups. Viral reactivation was a risk factor for 1-year OS (**Figure 1**). Further Cox regression analysis showed that viral infection was a risk factor affecting the 1-year OS (**Table 3**; HR, 12.625 for co-reactivation *vs*. no reactivation, P=0.021, and HR, 13.580 for CMV reactivation *vs*. no reactivation, P=0.013). However, viral reactivation was not a risk factor for LFS (**Figure 2**; p=0.348). The GVHD was a risk factor affecting the 1-year LFS (HR, 2.099 for GVHD *vs*. no GVHD, P=0.016). The 1-year OS and 1-year LFS hazards are summarized in **Table 3**.

**Table 3:** Risk factors for 1-year overall survival (OS) and 1-year leukemia-free survival (LFS).

Factors	OS		LFS	
	P value	HR:95%CI	P value	HR:95%CI
**Age**				
>40 years *vs*. <40 years	0.164	1.780 (0.790-4.009)	0.655	1.130 (0.661-1.933)
**Male *vs*. female**	0.663	1.199 (0.530-2.713)	0.792	1.070 (0.647-1.770)
**Underlying disease**				
AUL and CML	0.416	1.000	0.291	1.000
AML	0.934	—	0.923	—
ALL	0.931	—	0.924	—
MDS and AA	0.925	—	0.916	—
**Time from diagnosis to transplantation**				
More than 1 year *vs*. Less than 1 year	0.246	0.289 (0.035-2.357)	0.458	0.713 (0.292-1.741)
**Hemorrhagic cystitis**	0.313	1.477 (0.692-3.154)	0.761	1.086 (0.637-1.852)
**HLA match**				
Identical	0.863	1.000	0.947	1.000
Unrelated donor	0.746	0.691 (0.074-6.490)	0.993	1.006 (0.269-3.767)
Haploidentical	0.598	0.753 (0.263-2.158)	0.767	0.904 (0.464-1.762)
**GVHD**	0.288	0.605 (0.239-1.529)	**0.016**	2.099 (1.138-3.870)
**Neutrophil engraftment**	0.296	1.064 (0.947-1.196)	0.291	1.043 (0.965-1.127)
**Disease status**				
CR2 or NR *vs*. CR1	0.386	2.575 (0.303-21.903)	0.706	1.333 (0.299-5.945)
**CMV and EBV reaction conditions**				
No reactivation group	0.098	1.000	0.364	
Co-reactivation group	**0.021**	12.625 (1.464-108.874)	0.490	1.331 (0.591-3.001)
CMV reactivation group	**0.013**	13.580 (1.744-105.721)	0.096	1.742 (0.905-3.350)
EBV reactivation group	0.855	—	0.750	—

The meaning of the bold values are p < 0.05.

### Co-Reactivation of CMV and EBV Association With Risk Factors

Patients with co-reactivation of CMV and EBV were compared with all other patients to identify risk factors associated with co-reactivation. The competing risk model showed that age(>=40years *vs*. <40years)was one risk factor for co-reactivation of CMV and EBV (**Table 4**; HR:95%CI 1.360(1.008-1.833). Another risk factor was HLA match (HR:95%CI (4.442(1.710-11.535) for unrelated donor *vs*. Identical, P=0.002; HR:95%CI 5.178(3.333-8.047) for haploidentical *vs*. Identical, P=0.000). GVHD and disease status were excluded as a risk factor for CMV and EBV co-reactivation in the competing risk model(P>0.05).

**Table 4:** Risk factors for cytomegalovirus (CMV) and Epstein-Barr virus (EBV) co-reactivation.

Factors	HR:95%CI	P value
**Age(>=40years *vs*. <40years)**	1.360(1.008-1.833)	**0.044**
**Sex (Male *vs*. female)**	1.225(0.931-1.612)	0.146
**Underlying disease**		
AML *vs*. AUL and CML	1.267(0.635-2.529)	0.502
ALL *vs*. AUL and CML	1.335(0.680-2.623)	0.401
MDS and AA *vs*. AUL and CML	1.080(0.525-2.218)	0.835
**Time from diagnosis to transplantation**		
More than 1 year *vs*. Less than 1 year	0.877(0.495-1.553)	0.652
**Hemorrhagic cystitis**	1.311(0.973-1.766)	0.075
**HLA match**		
Unrelated donor *vs*. Identical	4.442(1.710-11.535)	**0.002**
Haploidentical *vs*. Identical	5.178(3.333-8.047)	**0.000**
**GVHD**	0.951(0.714-1.268)	0.735
**Neutrophil engraftment**	0.424(0.124-1.452)	0.172
**Disease status**		
CR2 or NR *vs*. CR1	1.689(0.854-3.340)	0.132

The meaning of the bold values are p < 0.05.

## Discussion

In our study, we proved that CMV and EBV co-reactivation in HSCT patients were associated with OS and LFS. Our findings are thus clinically relevant. CMV reactivation is known to be significantly associated with EBV reactivation (Fan et al., 2016), but CMV and EBV co-reactivation is relatively less widespread compared to that of the other double-stranded DNA viruses. In our study, CMV and EBV co-reactivation was found in 22.9% of patients after HSCT (n=75). This is consistent with a previous study that reported CMV-EBV coinfection, in which the rates of co-infection post-transplantation varied between 3.0% and 32.7% (Anderson-Smits et al., 2020). In the study reporting the lowest rate (3.0%,12/404), the total study population included patients who were not included in the groups at a high risk of EBV-related complications, in high-stakes patients, and in those who were not high stakes but had improved moderate-to-poor steroid-refractory GVHD (Garcia-Cadenas et al., 2015). Although the proportion of patients with CMV-EBV co-reactivation was reported with EBV-PTLD (post-transplant lymphoproliferative disorders), it is unclear whether this occurs for all cases of co-reactivation (i.e., CMV-EBV co-reactivation without PTLD), which might have helped to decrease the rate of co-reactivation. In the report citing the highest rate of CMV-EBV co-reactivation (32.7%; 33/101), all cases of co-reactivation involved both EBV and CMV (defined by viral load) (Zallio et al., 2013), and a similar definition of co-reactivation was used to that in our study. Another study reported CMV-EBV coinfection rates of 22.7% (10/44) (Fan et al., 2016) with a similar incidence of co-reactivation under the same definition.

Our study proved that CMV and EBV co-reactivation were related to a decreased 1-year OS. However, the causes of death (NRM or relapse) did not significantly differ among the groups (P>0.05). This result is partly in accordance with that of a previous study. Co-reactivation of CMV and EBV was found to be related to a decreased 1-year OS, which was mainly because of an increase in NRM (Zhou et al., 2020). With co-reactivation, the 1-year NRM was higher, in contrast to that found by two other groups, the difference was not remarkably significant (P=0.053), and no deaths occurred due to relapse (Zhou et al., 2020). Another report showed that a CMV and EBV co-reactivation group had a significantly higher 6-month non-relapse mortality than the other groups with CMV or EBV reactivation alone (Song et al., 2014). According to our findings, CMV reactivation alone after HSCT was also associated with 1-year OS; however, EBV reactivation alone after HSCT was not. One meta-analysis showed that CMV reactivation is associated with an increased risk of overall mortality and NRM in allo-HSCT recipients (Gimenez et al., 2019).

According to our results, the duration of CMV viremia was found in the co-reactivation group as compared to that in the other reactivation groups, reflecting the effect of EBV reactivation on CMV reactivation. CMV induces NKG2C + CD57 + KIR+ natural killer (NK) cell expansion 3–6 months after HSCT (Zuo and Zhao, 2021). NK cells are the earliest reconstituting immune cells, achieving normal numbers within weeks after patients undergo HSCT and helping in the graft-versus-tumor function along with T cells (Farag et al., 2002). Meanwhile, the cytokine-producing and cytotoxic functions of NK cells were found to be lower until 3–6 months after HSCT (Foley et al., 2011) and reached common reactivity levels at the first year and were maintained during later times (Haas et al., 2011). Immunoreactivation of one virus mediated by another virus has been previously reported with both HSCT and solid organ transplantation. This could also demonstrate poor immune reconstitution as previously shown with poor CD3+ and CD4+25+ cell counts (on day 30) (Zhou et al., 2020). CMV and EBV co-reactivation induces an increase in CD56dim/NKG2A+/CD57+NK cell numbers, which remain elevated up to 6 months after reactivation and leads to a decrease in the absolute quantity of immature CD56bright/CD16− NK cells in the blood (Lunemann et al., 2013; Hendricks et al., 2014). One study showed that CMV is an especially active inducer of some members of the herpesvirus family and implied that the interplay between CMV and EBV occurs unidirectionally *in vivo* (Aalto et al., 1998). However, this study was not based on HSCT patients.

Our study identified HLA match (unrelated donor and haploidentical *vs*. Identical) as an independent risk factor for CMV and EBV co-reactivation. Compared with HLA identical sibling transplantation, patients undergoing HLA-haploidentical stem cell transplantation(haploSCT) usually receive more intensive immunosuppressors to guarantee engraftment and later prevent graft-versus-host disease (GVHD) (Luo et al., 2021). Previous studies have shown that risk factors for CMV reactivation after HSCT include a donor or recipient seropositive for CMV, mismatched or unrelated donors, pre-allo-HSCT viremia, and use of alemtuzumab (Sousa et al., 2014). Another findings show patients undergoing hematopoietic stem cell transplantation or solid organ transplantation can experience post-transplant lymphoproliferative disorders due to dysfunction or suppression of host’s immune system, or uncontrolled proliferation of EBV-infected cells (Fujimoto and Suzuki, 2020). Some study uncovered a significant correlation of recovered Vδ2 with EBV reactivation following haploSCT (Liu et al., 2018). HSCT is a process comprising total immune reconstruction, and the interplay of various viruses in this process makes the condition of HSCT patients more complicated and diverse.

There are several limitations to this study. First, the retrospective nature of the study has inherent risks of bias; nevertheless, the patient characteristics and the HSCT complications did not differ significantly from those reported in prospective research. Second, we did not study other herpesviruses, which might have had a significant effect on these findings. However, we studied the two most clinically significant viruses that have defined treatment options. Despite these limitations, we believe that this study provides clinical hematologists with scientific evidence of CMV and EBV co-reactivation.

## Data Availability Statement

The raw data supporting the conclusions of this article will be made available by the authors, without undue reservation.

## Ethics Statement

The studies involving human participants were reviewed and approved by the Ethics Committee of the Peking University People’s Hospital. The patients/participants provided their written informed consent to participate in this study.

## Author Contributions

SL and MS: design of the study; SL and NZ: data acquisition, analysis, and interpretation; MJ: drafted the article and critically revised the manuscript; MS: gave final approval for the version to be submitted. All authors contributed to the article and approved the submitted version.

## Funding

This study was approved by the National Natural Science Foundation of China to MS (Grant No. 81870196).

## Conflict of Interest

The authors declare that the research was conducted in the absence of any commercial or financial relationships that could be construed as a potential conflict of interest.

## Publisher’s Note

All claims expressed in this article are solely those of the authors and do not necessarily represent those of their affiliated organizations, or those of the publisher, the editors and the reviewers. Any product that may be evaluated in this article, or claim that may be made by its manufacturer, is not guaranteed or endorsed by the publisher.
