# Supplementary material for: Association Between Cytomegalovirus and Epstein-Barr Virus Co-Reactivation and Hematopoietic Stem Cell Transplantation
Source: Front Cell Infect Microbiol. 2022 Mar 25;12:818167. doi: 10.3389/fcimb.2022.818167 (PMC8992791; doi:10.3389/fcimb.2022.818167)
Supplement: Supplementary file 1 [file Table_1.docx]

**Supplementary table S1**. Flow chart of patients included in the analysis

Abbreviations: PUIH, Peking University People’s Hospital, Institute of Hematology; HSCT, hematopoietic stem cell transplantation; CMV, Cytomegalovirus; EBV, Epstein-Barr virus

| patients | groups | mortality cause |
| --- | --- | --- |
| 1 | Co-reactivation group | sepsis |
| 2 | Co-reactivation group | bad transplantation；respiratory failure |
| 3 | Co-reactivation group | relapse |
| 4 | Co-reactivation group | pulmonary infection；respiratory failure；GVHD |
| 5 | Co-reactivation group | multiple organ failure |
| 6 | Co-reactivation group | pulmonary infection |
| 7 | Co-reactivation group | relapse |
| 8 | Co-reactivation group | relapse |
| 9 | Co-reactivation group | sepsis； GVHD |
| 10 | CMV reactivation group | pulmonary infection；respiratory failure |
| 11 | CMV reactivation group | sepsis； liver failure |
| 12 | CMV reactivation group | pulmonary infection；respiratory failure |
| 13 | CMV reactivation group | pulmonary infection |
| 14 | CMV reactivation group | relapse |
| 15 | CMV reactivation group | pulmonary infection；respiratory failure；GVHD |
| 16 | CMV reactivation group | pulmonary infection；respiratory failure；GVHD |
| 17 | CMV reactivation group | pulmonary infection；respiratory failure |
| 18 | CMV reactivation group | sepsis |
| 19 | CMV reactivation group | relapse |
| 20 | CMV reactivation group | pulmonary infection；multiple organ failure |
| 21 | CMV reactivation group | pulmonary infection |
| 22 | CMV reactivation group | liver failure |
| 23 | CMV reactivation group | relapse |
| 24 | CMV reactivation group | bone marrow failure； epilepsy； coma |
| 25 | CMV reactivation group | relapse |
| 26 | CMV reactivation group | pulmonary infection；respiratory failure |
| 27 | CMV reactivation group | multiple organ failure |
| 28 | CMV reactivation group | pulmonary infection；respiratory failure；GVHD |
| 29 | CMV reactivation group | relapse |
| 30 | CMV reactivation group | relapse |
| 31 | No reactivation group | relapse |
| 32 | No reactivation group | pulmonary infection |
| 33 | No reactivation group | respiratory failure；serious myocarditis |

**Supplementary table S2.** **Causes of mortality**

Abbreviations: CMV, Cytomegalovirus; GVHD, graft-versus-host-disease
